# Data on diversity and abundance of zooplanktons along the northern part of the Persian Gulf, Iran

**DOI:** 10.1016/j.dib.2018.06.012

**Published:** 2018-06-13

**Authors:** Azade Izadi, Sina Dobaradaran, Iraj Nabipour, Vahid Noroozi Karbasdehi, Ehsan Abedi, Hossein Darabi, Mohammad Ansarizadeh, Bahman Ramavandi

**Affiliations:** a Department of Environmental Health Engineering, Faculty of Health, Bushehr University of Medical Sciences, Bushehr, Iran; b The Persian Gulf Marine Biotechnology Research Center, The Persian Gulf Biomedical Sciences Research Institute, Bushehr University of Medical Sciences, Bushehr, Iran; c Systems Environmental Health, Oil, Gas and Energy Research Center, The Persian Gulf Biomedical Sciences Research Institute, Bushehr University of Medical Sciences, Bushehr, Iran; d The Persian Gulf Tropical Medicine Research Center, The Persian Gulf Biomedical Sciences Research Institute, Bushehr University of Medical Sciences, Bushehr, Iran; e Iranian National Institute for Oceanography and Atmospheric Science, Persian Gulf Center, Bushehr, Iran; f Department of Environmental Health Engineering, Sepidan Higher Educational Complex, Shiraz University of Medical Science, Shiraz, Iran

**Keywords:** Bushehr province, *Centropages spp.*, Persian Gulf, Zooplankton

## Abstract

In this data article, we aimed to evaluate and compare the biological diversity and relative abundance of zooplankton communities in 3 different areas along the northern part of the Persian Gulf in 3 different seasons. Data showed that *Centropages spp* and *Fish larvae* were the highest and lowest species among the groups identified in summer in Lavare Saheli and Nakhle Taghi with relative abundances of 87% and 2.7% respectively. In winter, *Cyphonautes larvae* and *Corycaeus spp.* were the highest and lowest species in Kangan and Lavare Saheli with relative abundances of 57.1% and 1.88%, respectively. Also *Decapoda larvae spp.* and *Gastropoda larvae* were the highest and lowest species in spring in Kangan with relative abundances of 62.5% and 4.7% respectively. Data may serve as benchmarks for other groups working in the field of pollution control, aquatic ecosystem, and toxicology.

**Specifications Table**

**Table:** 

*Subject area*	*Ecology*
*More specific subject area*	*Seawater ecology: The abundance of zooplankton in the Persian Gulf*
*Type of data*	*Table and figure*
*How data was acquired*	*Zooplanktons were identified by using a Nikon SMZ1500 (Japan) zoom stereomicroscope.*
*Data format*	*Raw and analyzed*
*Experimental factors*	*All* samples *were collected by using Bongo net (*300 μm *mesh) by surface tow and a constant speed of 2 knots during 5 *min*. For identification, each sample was condensed into a* 1* *l *plastic bottle by 96% alcohol stabilized and transferred to laboratory for further study.*
*Experimental features*	*Evaluate biological diversity and relative abundance of zooplankton communities in the northern part of the Persian Gulf.*
*Data source location*	*Bushehr, northern part of the Persian Gulf, Iran*
*Data accessibility*	*Data is with this article.*

**Value of the data**

• Data can be used as a base-line data for abundance of zooplankton communities in marine environments and understanding industrial activities effects on abundance of these organisms.
• Data shown here can be useful for policy makers, managers, and all related stakeholders, companies, agencies, and institutes working in the fields of environment by imposing proper measures to protect environment.
• Data shown here may serve as benchmarks for other groups working or studying in the field of pollution control, aquatic ecosystem, and toxicology.

## 1 Data

In the data, as shown in Table 1, Table 2, Table 3, taxon and relative abundance of zooplankton samples in different seasons are presented. The results indicated that among the groups identified, *Centropages spp* and *Fish larvae* were the highest and lowest species in summer in Lavare Saheli and Nakhle Taghi with relative abundances of 87% and 2.7% respectively. In winter, *Cyphonautes larvae* and *Corycaeus spp.* were the highest and lowest species in Kangan and Lavare Saheli with relative abundances of 57.1% and 1.88% respectively. Also resulted showed that *Decapoda larvae spp.* and *Gastropoda larvae* were the highest and lowest species in spring in Kangan with relative abundances of 62.5% and 4.7% respectively.

**Table 1:** The taxon and relative abundance (%) of zooplankton recorded at the studied stations in winter (maximum values are expressed as bold italics; minimum values as bold underlined).

**Zooplankton**	**S**_**1**_	**S**_**2**_	**S**_**3**_	**S**_**4**_	**S**_**5**_	**S**_**6**_	**S**_**7**_	**S**_**8**_	**S**_**9**_	**S**_**10**_	**S**_**11**_	**S**_**12**_	**S**_**13**_	**S**_**14**_	**S**_**15**_
***Copepoda***															
*Copepodites*	–	–	11.1	28.6	5.3	–	–	–	–	–	–	–	–	–	3.72
*Acrocalanus spp.*	–	–	–	–	–	15.2	18	51.9	45.3	7.14	16.1	31.8	45.5	7.15	29.6
*Acartia spp.*	–	–	–	–	–	–	10	7.4	9.43	–	–	4.54	6.04	–	7.45
*Temora spp.*	–	–	–	–	–	–	5	–	–	–	3.3	9.1	3.01	–	18.5
*Corycaeus spp.*	–	–	–	–	–	12.1	5	–	**1.88**	–	–	2.24	–	7.15	7.4
*Centropages spp.*	36.4	34.2	–	–	–	24.2	23	18.5	15.1	21.4	–	11.4	–	–	22.2
***Bivalvia larvae***	13.6	–	11.1	–	–	–	–	–	–	–	–	–	–	–	–
***Gastropoda larvae***	–	–	–	–	–	–	–	–	–	–	–	–	6.04	–	–
***Tunicata***															
*Oikopleura sp.*	–	–	33.4	28.6	45.3	–	–	–	3.77	–	–	–	–	–	–
***Polychaeta larvae***	–	–	–	–	–	–	–	–	1.89	7.18	–	–	–	–	–
***Chaetognatha***															
*Sagitta sp.*	–	–	–	14.2	11.2	6.06	2.5	–	1.89	–	–	2.28	–	–	–
***Bryozoa larvae***															
*Cyphonautes larvae*	31.8	40.8	22.2	14.3	–	27.3	20	14.8	11.3	***57.1***	51.6	18.2	9.1	14.3	7.41
***Cirripedia naupllii***	–		–	–	–	–	2.5	–	–	–	–	–	–	–	–
***Unidentified eggs***	9.12	10.38	–	14.3	38.2	9.09	7.5	–	–	–	–	4.54	3.01	–	3.72
***Medusa***	4.54	6.51	–	–	–	–	2.5	3.7	5.66	–	29	15.9	27.3	71.4	–
***Decapoda larvae***	–	–	–	–	–	–	5	3.7	1.9	7.18	–	–	–	–	–
***Radiolaria***	4.54	8.11	22.2	–	–	6.05	–	–	1.89	–	–	–	–	–	–

S_1–5_=Nakhle Taghi; S_6–10_=Kangan; S_11–15_=Lavare Saheli.

**Table 2:** The taxon and relative abundance (%) of zooplankton recorded at the studied stations in spring (maximum values are expressed as bold italics; minimum values as bold underlined).

**Zooplankton**	**S**_**16**_	**S**_**17**_	**S**_**18**_	**S**_**19**_	**S**_**20**_	**S**_**21**_	**S**_**22**_	**S**_**23**_	**S**_**24**_	**S**_**25**_	**S**_**26**_	**S**_**27**_	**S**_**28**_	**S**_**29**_	**S**_**30**_
***Copepoda***															
*Copepodites*	–	–	–	–	–	–	–	–	–	–	20	–	–	–	–
*Acrocalanus spp.*	27.3	9	30	18.2	–	–	12.5	14.3	–	12.5	20	–	16.7	14.3	25
*Temora spp.*	–	9.1	–	–	–	–	–	7.1	11.8	–	–	–	–	–	–
*Oithona sp.*	–	27.3	–	18.2	–	25	–	7.15	17.8	–	–	–	11.1	14.3	8.3
***Gastropoda larvae***	–	–	–	–	–	–	–	–	–	–	–	–	–	**4.7**	–
***Tunicata***															
*Oikopleura sp.*	–	–	–	9.1	–	–	–	–	–	–	–	–	–	–	–
***Polychaeta larvae***	–	–	5	–	–	–	–	7.1	–	–	–	8.33	–	–	–
***Chaetognatha***															
*Sagitta sp.*	–	–	–	9.1	12.5	–	–	–	–	–		–	–	–	–
***Unidentified eggs***	27.3	18.2	40	27.2	25	25	12.5	14.3	29.3	31.3	20	25	27.8	28.6	8.35
***Medusa***	18.2	18.2	15	9.1	12.5	25	12.5	7.15	5.8	12.6	20	8.34	5.5	4.8	8.33
***Decapoda larvae***	27.3	18.2	10	9.1	50	25	***62.5***	42.9	35.3	37.4	20	50	38.9	33.3	50
***Fish larvae***	–	–	–	–	–	–	–	–	–	6.2	–	8.33	–	–	–

S_16–20_: Nakhle Taghi; S_21–25_: Kangan; S_26–30_: Lavare Saheli.

**Table 3:** The taxon and relative abundance (%) of zooplankton recorded at the studied stations in summer (maximum values are expressed as bold italics; minimum values as bold underlined).

**Zooplankton**	**S**_**31**_	**S**_**32**_	**S**_**33**_	**S**_**34**_	**S**_**35**_	**S**_**36**_	**S**_**37**_	**S**_**38**_	**S**_**39**_	**S**_**40**_	**S**_**41**_	**S**_**42**_	**S**_**43**_	**S**_**44**_	**S**_**45**_
***Copepoda***															
*Acrocalanus spp.*	23	5.4	42.9	13	6	20	17	7.54	13	10.9	8.6	7.7	17	4.3	8.6
*Acartia spp.*	–	5.4	–	–	–	20	5	3.76	–	–	–	5.1	–	–	
*Centropages spp.*	46.2	81.1	42.9	63	75	50	55	71.7	75	70.5	74	74	67	***87***	82.3
*Labidocera sp.*	15.4	5.4	14.2	25	13	10	23	17	13	18.6	8.8	5.2	17	8.7	9.1
***Medusa***	–	–	–	–	–	–	–	–	–	–	4.3	–	–	–	–
***Decapoda larvae***	–	–	–	–	–	–	–	–	–	–	4.3	5.1	–	–	–
***Fish larvae***	15.4	**2.7**	–	–	6	–	–	–	–	–	–	2.9	–	–	–

S_31–35_: Nakhle Taghi; S_36–40_: Kangan; S_41–45_: Lavare Saheli.

## 2 Experimental design, materials and methods

### 2.1 Study area description

**Fig. 1:**
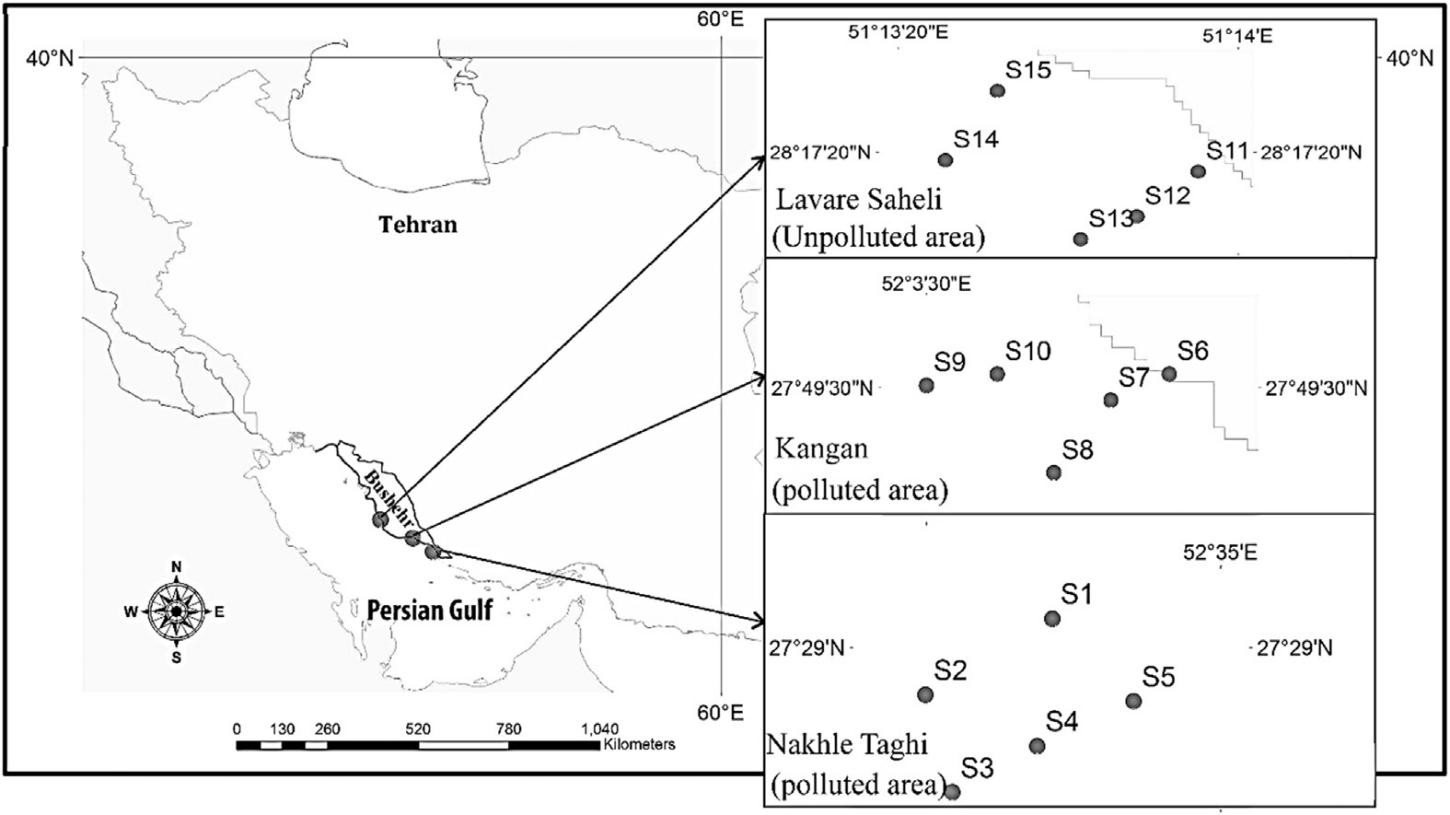
The geographical location of zooplankton samples from 3 points along the northern part of Persian Gulf map and locations of sampling stations.

Three different areas were selected in the northern part of the Persian Gulf, Bushehr province, Iran as sampling points including Nakhle Taghi, Kangan and Lavar-e-Saheli (Fig. 1). Features of the northern part of the Persian Gulf are shallow, limited circulation and high salinity [1]. The time of water turnover in the basin is between 3 and 5 years and shows that pollutants likely to reside in the Persian Gulf for a significant time [2], this has caused the north parts of the Persian Gulf to be much more influenced by contaminations [1]. Also, the former studies in the northern part of the Persian Gulf showed that these areas are affected by pollution [3], [4], [5], [6], [7].

### 2.2 Sample collection

Samples were collected from Mar to Aug 2016, at 3 different locations and 5 sampling stations (15 samples from each station) approximately every 2 months along the Persian Gulf in the Bushehr port coastal area. Zooplankton samples were collected by using a Bongo net (mesh: 300 μm) by surface pull and performed at a constant speed (2 knots) during 5 min. Exact coordinates of sampling points are shown in Table 4.

**Table 4:** Location of zooplankton samples from 3 locations along the Persian Gulf in the Bushehr province.

**Region**	**Site no.**	**Location**
Nakhle Taghi	1	N27°29′02.12′′ E052°34′47.41′′
Nakhle Taghi	2	N27°28′56.48′′ E052°34′37.96′′
Nakhle Taghi	3	N27°28′49.32′′ E052°34′39.98′′
Nakhle Taghi	4	N27°28′52.76′′ E052°34′46.32′′
Nakhle Taghi	5	N27°28′56.01′′ E052°34′53.51′′
Kangan	1	N27°49′31.23′′ E052°03′51.59′′
Kangan	2	N27°49′28.87′′ E052°03′46.35′′
Kangan	3	N27°49′22.52′′ E052°03′41.33′′
Kangan	4	N27°49′30.21′′ E052°03′29.97′′
Kangan	5	N27°49′31.23′′ E052°03′36.28′′
Lavare Saheli	1	N28°17′17.49′′ E051°13′55.32′′
Lavare Saheli	2	N28°17′11.67′′ E051°13′48.12′′
Lavare Saheli	3	N28°17′08.73′′ E051°13′41.94′′
Lavare Saheli	4	N28°17′18.95′′ E051°13′25.55′′
Lavare Saheli	5	N28°17′27.94′′ E051°13′31.67′′

### 2.3 Identification of genus and species of zooplankton

For identification, each sample was condensed into a 1 l plastic bottle by 96% alcohol stabilized and transferred to laboratory. Zooplanktons were enumerated and identified by using a Nikon SMZ1500 (Japan) zoom stereomicroscope and Zooplankton identification guide.
